# Gemcitabine-Induced Myonecrosis Following Hypofractionated Radiation

**DOI:** 10.7759/cureus.58591

**Published:** 2024-04-19

**Authors:** Merav A Ben-David, Ignat Schwartz, Iris Eshed, Keren Levanon

**Affiliations:** 1 Department of Oncology, Assuta Medical Center, Tel Aviv, ISR; 2 Faculty of Health Sciences, Ben Gurion University, Beer-Sheva, ISR; 3 Department of Pathology, Sheba Medical Center, Ramat Gan, ISR; 4 Department of Diagnostic Imaging, Sheba Medical Center, Ramat Gan, ISR; 5 School of Medicine, Faculty of Medical and Health Sciences, Tel Aviv University, Tel Aviv, ISR; 6 The Jusidman Cancer Center, Sheba Medical Center, Ramat Gan, ISR

**Keywords:** radiation-induced myonecrosis, hypofractionation, myositis, gemcitabine, radiation recall phenomenon

## Abstract

Palliative radiation is often used to abate pain and prevent bone fractures in patients with metastatic cancer. Hypofractionation, meaning delivery of larger doses of radiation in each treatment session (fraction), has become the standard of care in most cases. It not only reduces the burden on the medical system and facilitates the relief of symptoms but also enables the maintenance of the continuity of systemic therapy. Radiation recall phenomenon (RRP) is an acute inflammatory reaction in previously irradiated tissues that is provoked by chemotherapeutic drug administration. The incidence, severity, and prognosis of RRP following hypofractionated radiation therapy have not been studied. The symptoms of RRP depend on the radiation field, with the greatest concern associated with mucosal and dermal damage, though other symptoms have also been reported. Here, we describe a case of a 41-year-old woman with metastatic breast cancer (hormone receptor-positive, HER2/neu negative), who received palliative radiation to four other fields along the course of her disease, before her presentation with isolated myonecrosis of the thigh muscles. This RRP occurred four months following the last of two fractions of 8 Gy radiation to this region, given three months apart, and after six courses of cisplatin + gemcitabine. The symptoms improved with cessation of gemcitabine and prolonged administration of non-steroidal anti-inflammatory medications.

## Introduction

Radiation recall phenomenon (RRP) is an inflammatory condition developing in previously irradiated fields within days to several weeks following exposure to a provoking systemic chemotherapeutic agent [1]. This relatively uncommon event has been previously reported with a range of cytotoxic agents, including gemcitabine [2], a widely used antimetabolite nucleoside analog. RRP attributed to gemcitabine tends to affect visceral organs more than the skin (70% of cases) [3]. This differs from the classic dermatitis reported in the majority of cases of RRP attributed to other cytotoxic agents. Moreover, gemcitabine-induced visceral RRP is associated with a short interval between the end of radiation treatment and the exposure to systemic therapy. In this case report, we described isolated myositis and myonecrosis as a result of gemcitabine-induced RRP in a young breast cancer patient. This patient had been previously treated with palliative radiation to several body parts and also received whole brain radiation therapy (WBRT) shortly before the administration of gemcitabine. However, the patient developed severe RRP only in a single radiation field, which was irradiated twice with single fractions of 8 Gy each. Although skeletal muscles are relatively resistant to radiation injury [4,5], a retrospective analysis indicates that increased total dose and hypofractionation (irradiation with fewer, larger doses compared to conventional radiation in 1.8-2 Gy fractions) increased the likelihood of developing radiation-induced muscle injury [6]. This case highlights the increased risk for gemcitabine-induced RRP in body organs that were exposed to hypofractionated radiation.

## Case presentation

A 41-year-old woman was diagnosed with metastatic, hormone receptor-positive, HER2/neu negative breast cancer five years before the reported event. She underwent multiple lines of endocrine, biological, and chemotherapies, as well as denosumab, and experienced progression while receiving vinorelbine treatment. Along the course of her disease, she received fractionated radiation to the right and left pelvic bones spine (each treated to 20 Gy in five fractions), right acetabulum (single fraction of 8 Gy), sacroiliac joint (single fraction of 8 Gy), and whole brain (10 fractions of 3 Gy to a total dose of 30 Gy).

**Figure 1:**
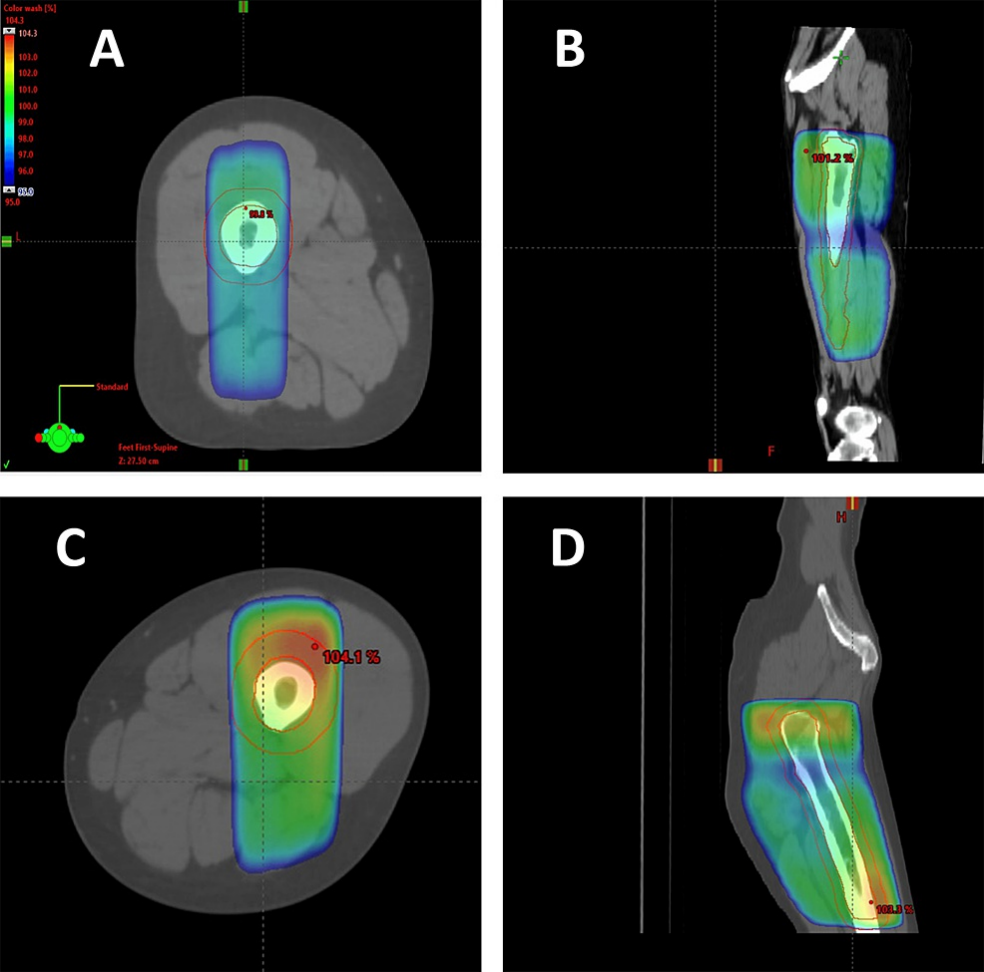
Radiation fields to the left femur 95% isodose: (A) axial view, recent treatment; (B) sagittal view, recent treatment; (C) axial view, previous treatment; and (D) sagittal view, previous treatment.

Seven months before the presentation, she underwent treatment in a supine position with AP-PA fields to the femur, using 3D planning and calculation, with a single fraction of 8 Gy using 15X MV energy. The maximal point dose (dose max) was 104.4%. Relief of pain was substantial, though short-lasting. A second treatment was delivered two months later (five months before presentation), again in a supine position with 3D planning and calculation (however, in a slightly different setting inside the linear accelerator). She received 8 Gy using the same energy, again with AP-PA fields (Figure 1). The maximal point dose (dose max) was 104.3%. The second treatment was more effective in controlling the pain.

Two months after the second treatment of the left femur, she began systemic chemotherapy with cisplatin (25 mg/m²) and gemcitabine (650 mg/m²) on days 1 and 8, with cycles repeating every 21 days. Granulocyte colony-stimulating factor (G-CSF) was administered after each treatment due to anticipated neutropenia. A fluorodeoxyglucose positron emission tomography (FDG-PET) scan after six treatments indicated partial response in all disease sites.

Shortly after the sixth treatment, she began complaining about swelling and pain in the left upper leg. She underwent a Doppler ultrasonic evaluation of the limb, which excluded deep vein thrombosis. The patient self-administered low-dose dexamethasone for pain management.

She then underwent contrast-enhanced CT of the leg, revealing a hypodense lesion within the left semitendinosus muscle (density 24 HU) with circumferential enhancement. Additionally, adipose tissue infiltration was observed around the posterior and anterior compartments of the leg, along with infiltration along the superficial and deep fascias, and along the femoral vessels. Laboratory blood results showed leukocytosis (probably steroid-induced) and C-reactive protein (CRP) within the normal range. A follow-up ultrasound of the soft tissues two days later excluded abscess formation but showed edema of the hamstring muscles. At this point, CRP was elevated, and the patient was admitted for analgesic and antibiotic treatment for suspected fasciitis and/or myositis. Her condition did not improve.

**Figure 2:**
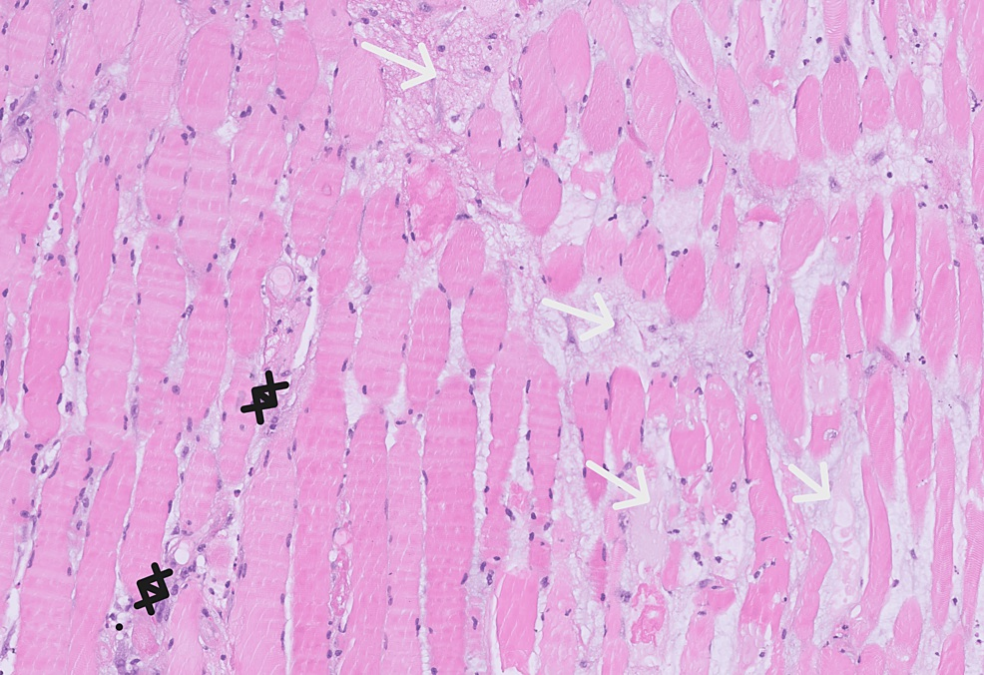
Hematoxylin and eosin slide of semitendinosus muscle biopsy. The biopsy indicates acute inflammation, characterized by infiltration of neutrophils, lymphocytes, and macrophages (black markers), and diffuse prominent interstitial edema (white arrows), indicated by fluid within the extracellular spaces of muscle tissue disrupting the arrangement of muscle fibers.

The patient then underwent a CT-guided biopsy from the left semitendinosus muscle. Histology showed cores of skeletal muscle with interstitial edema, disruption of muscle fiber architecture, acute inflammation with neutrophilic, lymphocytic, and macrophagocytic infiltration, and small foci of necrosis (Figure 2). Immunostains were negative for epithelial cells, excluding the possibility of breast cancer metastasis (not shown).

**Figure 3:**
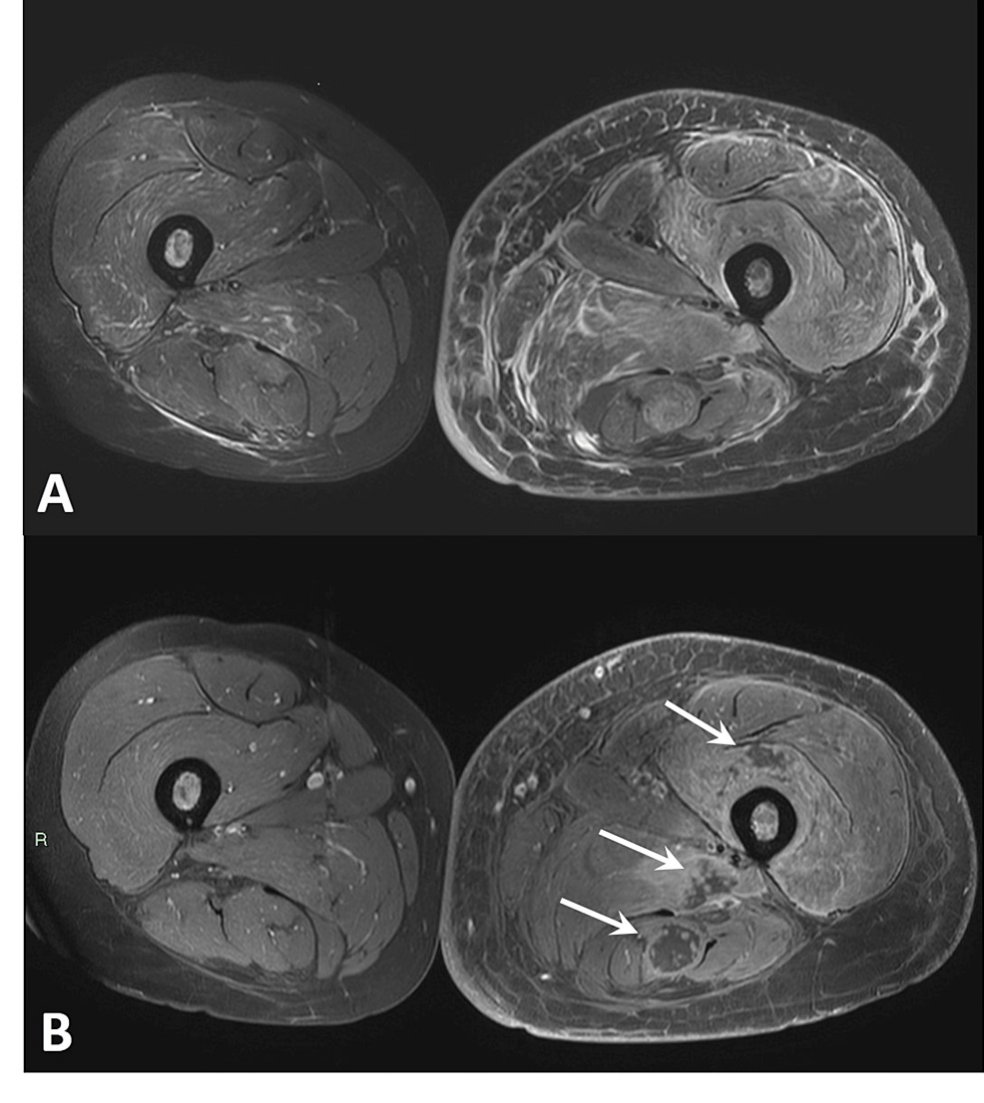
Magnetic resonance imaging of the thigh. (A) Axial T2-W with fat suppression and (B) T1-W with fat suppression after intravenous (IV) gadolinium injection MRI images of the thighs. Massive muscle edema involving the majority of the muscles of the left thigh is observed. Following contrast injection, multiple round myonecrotic lesions (arrows) are seen along the tract of the radiation beam.

The patient received another treatment of cisplatin-gemcitabine with further worsening of the pain and leg swelling. Gadolinium-enhanced MRI of the limb demonstrated sub-cortical and intramedullary avascular necrotic regions in the femur, edema of the subcutaneous adipose tissue, and diffuse edema of all the muscles extending from the gluteal region along the leg (Figure 3). Vast, continuous areas within the vastus intermedius and the hamstring muscles were indicative of necrosis, which raised suspicion for radiation-induced myonecrosis, probably triggered by exposure to gemcitabine.

Gemcitabine was stopped, and only cisplatinum was continued. The patient received a long course of non-steroidal anti-inflammatory drugs (NSAIDs) and improved after three weeks. No other previously irradiated body regions were affected by gemcitabine-induced RRP, including the brain, for which she received 10 fractions of WBRT (3 Gy/fraction) only a few days before initiation of cisplatin-gemcitabine.

## Discussion

Hypofractionation, or larger doses of radiation delivered over a shorter period, induces unique cellular effects compared to regular fractionation of radiation therapy (typically 1.8-2 Gy per fraction). These effects can improve the ablative effect over resistant tumor cells and overcome hypoperfusion and hypoxic conditions within tumors, without significantly increasing the the risk of acute and late toxicities in surrounding normal tissues [7]. Multiple randomized trials have shown the equivalent utility of single dose versus conventional fractionation palliative radiation therapy, in terms of pain control, with increased re-irradiation rate [8]. While the relationship between radiation doses and RRP is not fully elucidated, higher radiation doses, larger irradiated volumes, specific chemotherapy agents (specifically gemcitabine), and the timing of chemotherapy administration relative to radiation therapy are all factors that may influence its occurrence [1]. Gemcitabine is implicated in visceral RRP, rather than dermatitis, as seen in this case as well. However, in our case, the most recently irradiated field, which is the brain, did not develop any involvement. RRP symptoms following WBRT are extremely rare, most commonly attributed to gemcitabine [9,10], and occur in patients who have received higher doses of both radiation and chemotherapy than our patient. Achieving comparability of different fractionation schemes, such as those delivered to the thigh and the brain, can be accomplished by evaluating the equivalent dose in 2 Gy fractions (EDQ2) and the biologically effective dose (BED) for each treatment. In this case, utilizing a healthy brain's α/β ratio of 12, the EDQ2 was 32.14 and BED was 37.5 for WBRT administered at a dose of 30 Gy in 10 fractions. This is compared to the skeletal muscle's α/β ratio of four, with 8 Gy delivered twice, resulting in an EDQ2 of 32 and a BED of 48 (and probably lower due to the two-month gap between doses). It is plausible that the relatively low concentration of gemcitabine in the central nervous system (CNS), because of limited permeability through the blood-brain barrier is the reason for the lack of neurological manifestations in this case. Blood-brain barrier integrity has been maintained despite recent WBRT.

## Conclusions

This case raises the hypothesis that repeated high-dose, single-fraction radiation followed by gemcitabine administration is the cause of the severe RRP, which manifests as myonecrosis. The radiation inflicted tissue or vascular damage and diminished tissue repair capacity in the irradiated thigh muscles, while gemcitabine administration precipitated isolated reactivation of inflammatory response. The most recently irradiated field, the brain, was unaffected, despite a similar biologically effective dose, probably due to the protective effect of the blood-brain barrier.
